# Novel Population Pharmacokinetic Model for Linezolid in Critically Ill Patients and Evaluation of the Adequacy of the Current Dosing Recommendation

**DOI:** 10.3390/pharmaceutics12010054

**Published:** 2020-01-09

**Authors:** Amaia Soraluce, Helena Barrasa, Eduardo Asín-Prieto, Jose Ángel Sánchez-Izquierdo, Javier Maynar, Arantxazu Isla, Alicia Rodríguez-Gascón

**Affiliations:** 1 Pharmacokinetics, Nanotechnology and Gene Therapy Group, Faculty of Pharmacy, Centro de Investigación Lascaray-ikergunea, University of the Basque Country UPV/EHU, 01006 Vitoria-Gasteiz, Spain; amaiasoraluce@gmail.com (A.S.); arantxa.isla@ehu.eus (A.I.); 2 Intensive Care Unit, Araba University Hospital, 01004 Vitoria-Gasteiz, Spain; helenabarrasa@gmail.com (H.B.); fjmaynar@gmail.com (J.M.); 3 Pharmacometrics & Systems Pharmacology Research Unit, Department of Pharmacy and Pharmaceutical Technology, School of Pharmacy and Nutrition, University of Navarra, 31008 Pamplona, Spain; easin@unav.es; 4 Intensive Care Unit. Doce de Octubre University Hospital, 28041 Madrid, Spain; jasiruci@gmail.com

**Keywords:** linezolid, population pharmacokinetics, critically ill, continuous renal replacement therapies, pharmacokinetic/pharmacodynamics, continuous infusion

## Abstract

Antimicrobial treatment in critically ill patients remains challenging. The aim of this study was to develop a population pharmacokinetic model for linezolid in critically ill patients and to evaluate the adequacy of current dosing recommendation (600 mg/12 h). Forty inpatients were included, 23 of whom were subjected to continuous renal replacement therapies (CRRT). Blood and effluent samples were drawn after linezolid administration at defined time points, and linezolid levels were measured. A population pharmacokinetic model was developed, using NONMEM 7.3. The percentage of patients that achieved the pharmacokinetic/pharmacodynamic (PK/PD) targets was calculated (AUC_24_/MIC > 80 and 100% T_>MIC_). A two-compartment model best described the pharmacokinetics of linezolid. Elimination was conditioned by the creatinine clearance and by the extra-corporeal clearance if the patient was subjected to CRRT. For most patients, the standard dose of linezolid did not cover infections caused by pathogens with MIC ≥ 2 mg/L. Continuous infusion may be an alternative, especially when renal function is preserved.

## 1. Introduction

Linezolid is an antibiotic with activity against mycobacteria and most Gram-positive bacteria, such as staphylococci, streptococci, and enterococci. It has shown activity toward methicillin-resistant *Staphylococcus aureus* (MRSA) and vancomycin-resistant enterococci (VRE) [1]. Linezolid is commonly employed in intensive care units (ICU), to manage pneumonia, skin and soft tissue infections, or bacteremia, since about 50% of bloodstream infections in critically ill patients are caused by Gram-positive bacteria, which are frequently multi-drug-resistant strains (e.g., MRSA and VRE) [1,2].

Selecting the optimal antimicrobial posology becomes a challenge when treating infections in critically ill patients. On the one hand, they are commonly subjected to aggressive medical interventions, such as mechanical ventilation or renal replacement techniques. On the other hand, several pathophysiological alterations may contribute to changes in the volume of distribution (V) or in the clearance (CL) (e.g., acute kidney injury or hypoalbuminemia). Moreover, drugs administered to these patients are likely to have higher pharmacokinetic (PK) variability, and, therefore, it becomes difficult to select the optimal dose, leading to suboptimal patient outcomes in some cases [3].

Acute kidney injury (AKI) is one of the most common pathological conditions in the ICU, ranging from 30% to 60% of hospitalized subjects. Continuous renal replacement therapies (CRRT) are frequent procedures for AKI treatment, estimating that around 5% of the ICU patients undergo these techniques [4,5]. CRRT involve pumping blood from the patient’s circulation through an extracorporeal circuit incorporating a synthetic membrane. Plasma water and drugs with small molecular weight, small volume of distribution, and low protein binding are significantly removed by CRRT. Other factors that affect the elimination of drugs are the permeability of the membrane, the effluent flow, or the CRRT modality itself. All of them contribute to the considerable variability observed in antibiotic concentrations in critically ill patients with CRRT [6,7]. This fact should be taken into account for population pharmacokinetic model construction, which should evaluate the influence of extracorporeal clearance (CL_EC_) on the total body clearance of drugs [8]. Since many antibiotics are significantly eliminated by CRRT, the contribution of the extracorporeal elimination should be evaluated to minimize the risk of underdosing and, therefore, therapeutic failure or emergence of resistance. Regarding linezolid, although only around 30% is excreted unchanged in urine, it has low protein binding (30% in healthy volunteers) and a low molecular weight (337.45 g/mol); thus, it is likely to be eliminated by CRRT, especially with high-flux membranes [9].

The aim of this study was to develop a population pharmacokinetic model for linezolid in critically ill patients, including patients undergoing CRRT and patients with preserved renal function. A secondary objective was to know if the pharmacokinetic/pharmacodynamic targets indicative of efficacy are achieved after the administration of the standard dose (600 mg/12 h, as a 30-min infusion).

## 2. Materials and Methods

### 2.1. Study Population and Design

An open-label prospective multicenter study was performed in the ICU of three Spanish University hospitals: Araba (Vitoria-Gasteiz), Doce de Octubre (Madrid), and Joan XXIII (Tarragona). The protocol was approved by the Basque Clinical Research Ethics Committee (EPA2014025, 26 August 2015) and the Spanish Agency of Medicinal Products and Medical Devices (FJM-LIN-2012-01, 30 August 2012). All subjects, or their legal representatives, had previously signed a written informed consent. Patients were eligible for inclusion if (i) they were admitted to the ICU; (ii) they suffered from an infection probably caused by a Gram-positive microorganism and, therefore, were treated with linezolid; (iii) they gave informed consent; and (iv) it was possible to obtain plasma and effluent samples from the extracorporeal device when undergoing CRRT. Effluent samples were collected to calculate the fraction of linezolid eliminated through the extracorporeal circuit (sieving coefficient, Sc).

The exclusion criteria were age < 18 years, pregnancy, hypersensitivity to linezolid or any of the excipients, and being on any medicinal product which inhibits monoamine oxidase A or B.

Table 1 displays demographic and biochemical data for the patients. Creatinine clearance (Clcr) was estimated for each patient by using the following equation: Clcr (mL/min) = (Cru × Vu)/(Crp ×600 min), where Crp was the plasma creatinine concentration (mg/dL), Cru was the concentration of creatinine in urine (mg/dL), and Vu was the total urine volume (mL) collected in 10 h.

The fraction of the drug eliminated across the membrane of the extracorporeal circuit during CRRT, known as sieving coefficient (Sc), was calculated as the ratio of the linezolid area under the effluent concentration curve to the area under the plasma concentration curve over the dosage interval. The Sc was used to estimate the CL_EC_ as follows: CL_EC_ = Sc × Qef, where Qef is the effluent flow.

### 2.2. Drug Administration, Sampling Procedure, and Linezolid Quantification

Each patient was administered 600 mg of linezolid every 12 h, by intravenous infusion, over 30 min, except one subject, who received an infusion of 60 min. A mean of 8 doses was administered before sample collection, to ensure steady-state concentrations. Blood samples were drawn at pre-dose, at the end of infusion, and at 1, 2, 3, 6, 8 to 10 and 12 h. For patients undergoing CRRT, effluent samples were taken at the same time points.

Concentrations of linezolid in plasma and effluent samples were quantified, using high-performance liquid chromatography (HPLC) assays with ultraviolet detection. Plasma sample preparation consisted of a protein precipitation step with acetonitrile, where internal standard (propyl-4-hydroxybenzoate) had been previously diluted. Afterward, the samples were centrifuged (10 min at 12,000 rpm), and the supernatants were injected into the HPLC system. Effluent samples were directly injected into the system and analyzed. Separation was performed on a Simetry^®^ C8 column (4.6 × 150 mm × 5 µm). The mobile phase consisted of ammonium phosphate 0.5%: acetonitrile (66:34, *v*:*v*). Linearity range was established from 0.5 to 50 µg/mL for plasma samples and from 0.2 to 30 µg/mL for effluent samples. The analytical method was previously validated following the FDA [10] and EMA [11] guidelines. Both intra- and inter-day accuracy and precision assays were settled at the limit of quantification (0.5 and 0.2 µg/mL for plasma and effluent samples, respectively), and at three concentration levels in the range of the expected concentrations, 1.5, 10, and 40 µg/mL for plasma, and 0.6, 5, and 24 µg/mL for the effluent samples. The calculated concentration did not deviate more than 15% from nominal concentration. Moreover, intra- and inter-day coefficient of variation was never above 15%. Linezolid drug substance for standards and quality control was kindly provided by Pfizer Inc. (New York City, NY, USA, Pfizer Compound Transfer Program).

### 2.3. Population Pharmacokinetic Model

#### 2.3.1. Base Model

Nonlinear mixed-effects modeling was implemented by NONMEM 7.3 (ICON Clinical Research LLC, North Wales, PA, USA), to estimate linezolid PK population parameters, using first-order conditional estimation method with interaction. Drug concentrations were logarithmically transformed. The model selection was based on the decrease in objective function value (OFV), the relative standard errors (RSE) of the parameters, and the goodness-of-fit (GOF) plots. Residual error was shaped, and inter-individual variabilities (IIV) and possible covariance were also explored.

#### 2.3.2. Covariate Selection

Once the base model was selected, and in order to describe the IIV, all variables presented in Table 1 were studied as potential covariates. For patients treated with CRRT, CL_EC_ was considered as a fixed value per patient, calculated individually as Q_ef_ multiplied by the Sc. The other three dichotomous covariates were created, depending on whether the patients had one, two, or three of the following parameters out of the normal range: bilirubin (normal range: 0.3–1.9 mg/dL), GOT (normal range: 8–30 UI/L for males and 6–25 UI/L for females), and GPT (normal range: 8–35 UI/L for males and 6–25 UI/L for females).

Stepwise covariate model building was used (SCM tool in PsN 4.7.0) to identify covariate candidates. During the forward inclusion and backward deletion, *p* < 0.05 and *p* < 0.01 levels of significance were used, respectively. GOF plots were also used to confirm whether different models performed satisfactorily.

#### 2.3.3. Model Evaluation

GOF plots assessed were the individual and population predictions versus observations, the conditional weighted residuals (CWRES) against time after dose, and the individual weighted residuals (IWRES) versus individual predictions. Moreover, a VPC was built to explore the performance of the selected model (VPC tool in PsN 4.7.0). Thus, the 2.5th, 50th, and 97.5th percentiles for observed data were plotted. Afterward, a 1000-sample dataset was simulated from the final model parameter estimates, and the 95% confidence intervals for the simulated 2.5th, 50th, and 97.5th percentiles were represented for visual inspection, using the xpose4 package in R 3.4.0. Additionally, parameter precision was evaluated by performing a 2000-dataset bootstrap (Bootstrap tool in PsN 4.7.0).

#### 2.3.4. External Validation

A new group of patients (*n* = 11) was used for external validation, which was also used to elucidate if the administration of linezolid as a continuous infusion would lead to an improvement of the pharmacodynamic target attainment. Forty-four plasma samples from these patients were analyzed. Table 2 shows demographic and biochemical data of the patients, as well as the APACHE II health score. Patients of this group did not receive CRRT, and their data were compared with that of the patients used for the development of the model. For continuous covariates, a t-test or Mann–Whitney U test was performed, regarding whether they were normally distributed or not. Concerning categorical data, Pearson’s chi-squared test (χ^2^) was applied. IBM SPSS statistic^®^ software (version 21) was used.

Patients included in the external validation study were administered 1200 mg of linezolid per day by continuous intravenous infusion, after a 600 mg loading dose over 30 min. One plasma sample per day was withdrawn over four consecutive days, and linezolid concentrations were quantified by using the HPLC technique explained in Section 2.2.

For these patients, linezolid concentrations were predicted by using the developed PK model. Five thousand subject simulations were performed for the following Clcr values: 40, 80, 120, 160, 200, and 240, which included Clcr values observed in the study population. The 2.5th, 50th, and 97.5th percentiles of the concentrations were calculated for the simulated data and were compared against the observed data.

### 2.4. PK/PD Analysis in the Study Population

Linezolid presents concentration and time-dependent activity [12]. For the antibiotics with this activity profile, the time (expressed as percent of the dosing interval) that the plasma concentration is maintained above the minimum inhibitory concentration (%T > _MIC_), and the ratio of area under the concentration–time curve over 24 h to the MIC (AUC_24_/MIC) are the PK/PD indexes that best predict clinical efficacy [12]. AUC_24_/MIC > 80 and 100% T_>MIC_ were selected as targets indicative of efficacy [12]. Based on the measured concentrations, we calculated the percentage of patients with AUC_24_/MIC > 80 and 100% T_>MIC_ for MIC values of 1, 2, and 4 mg/L (probability of target attainment or PTA). The selected PK/PD target when linezolid was administered as continuous infusion was the ratio of the drug concentration at steady state to the MIC (C_ss_/MIC, equivalent to 100% T_>MIC_). Statistical analyses were performed by using IBM^®^ SPSS^®^ Statistics for Windows, Version 25. A *p*-value < 0.05 was considered statistically significant.

## 3. Results

A total of 40 critically ill patients were included to develop the pharmacokinetic model, and a total of 311 plasma samples were analyzed. The source of infection was pulmonary in 14 cases, abdominal in 10, neurological in nine, and biliary in two, with other sources in the other five cases.

When subjected to CRRT (*n* = 23), eight effluent samples per individual were collected. Eighteen patients underwent continuous venovenous hemodiafiltration (CVVHDF), and five subjects received venovenous hemodialysis (CVVHD). The effluent flow was set at a range from 1.1 to 3.3 L/h. Fluid balance was prescribed according to clinical status.

### 3.1. Population Pharmacokinetic Model

#### 3.1.1. Base Model

Linezolid plasma concentrations were best described by a two-compartment model. A statistically significant drop of the OFV was obtained with respect to the one-compartment fit (ΔOFV = −113.167). IIV of the CL and the central compartment volume of distribution (V_1_) was included, and no correlation between these two parameters was detected. Variability was modeled by using an exponential model for IIV, and a combined error model for the residual variability was found to be the most suitable.

#### 3.1.2. Covariate Selection

The linezolid CL was estimated as the sum of a non-renal component (CL_NR_), a renal component (CL_R_) conditioned by the Clcr, and the CL_EC_ if the patient underwent CRRT. The inclusion of Clcr in the model led to a reduction of the IIV of the CL, from 98.7% to 61.5% (ΔOFV = −30.451). CL_EC_ was considered as a fixed value per patient. Bilirubin, GOT, and GPT were grouped and studied as potential covariates; however, they were excluded from the final model, since a better fit was not obtained. Although patients with Clcr > 130 mL/min showed a higher apparent volume of distribution of the peripheral compartment (V_2_), in the final model, the Clcr was not included as a covariate of this PK parameter, since no improvement in the predicted concentrations was observed, and higher estimation errors were obtained. No other covariate resulted in a relevant reduction in the OFV.

#### 3.1.3. Model Evaluation

**Figure 1:**
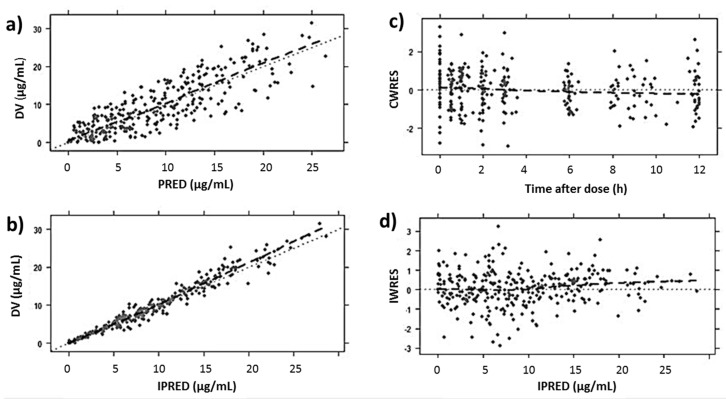
GOF plots obtained for the final model: (**a**) population predictions (PRED) and (**b**) individual predictions (IPRED) against observed linezolid plasma concentrations (DV, µg/mL), (**c**) conditional weighted residuals (CWRES) versus time after dose (h), and (**d**) the individual weighted residuals (IWRES) versus individual concentration predictions (µg/mL).

**Figure 2:**
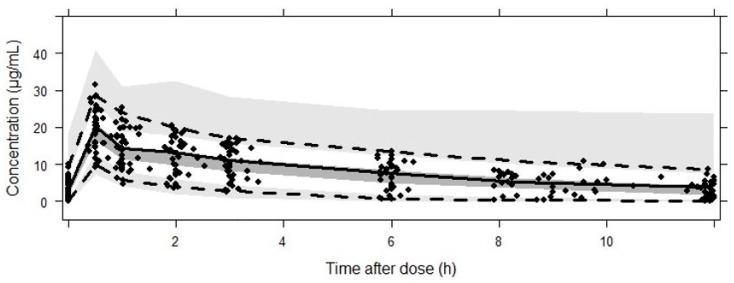
Results from the VPC from 0 to 12 h after dose. Dots correspond to the observed concentrations (µg/mL). The continuous line represents the median, while the dashed lines correspond to the 2.5th and 97.5th observed percentiles. Simulation-based 95% CIs for the median and both 2.5th and 97.5th percentiles are displayed by dark-gray and light-gray shading, respectively.

Table 3 shows the estimated parameter values of linezolid according to the PK population model and their RSE (%). Bootstrap results revealed that all parameters were estimated precisely. GOF plots obtained with the final model (Figure 1) showed no trend in CWRES or IWRES over time or predicted concentrations of the drug, respectively. Moreover, there was a good correlation between observed and predicted concentrations for both individual and population data. On the other hand, the VPC (Figure 2) showed a good correlation between raw data and the confidence intervals obtained by simulation.

#### 3.1.4. External Validation

**Figure 3:**
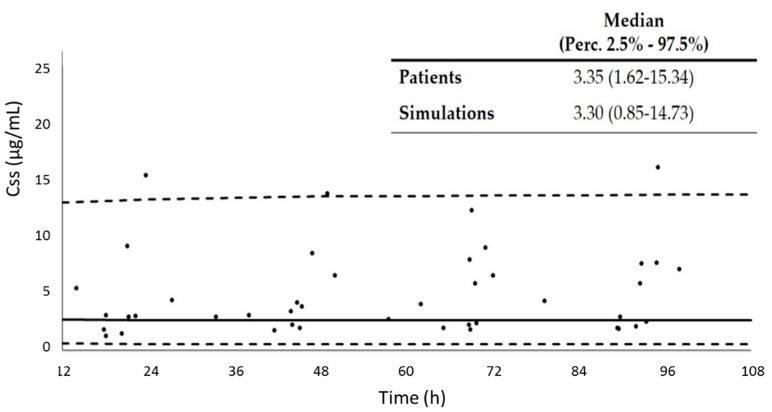
Comparison of the concentrations at steady-state (Css) observed in the patients used for the validation of the model, and the median value and 2.5% and 97.5% percentiles obtained by simulation, using the final model. Dots: experimental concentrations; continuous line: median of the simulated concentrations; dashed lines: 2.5th and 97.5th percentiles of the simulated concentrations.

Figure 3 shows the comparison of the concentrations at steady-state (Css) observed in the 11 patients used for validation, which received linezolid by continuous infusion, and the median value and 2.5% and 97.5% percentiles obtained by simulation, using the final model. As can be appreciated, our model was able to predict patients’ concentrations properly.

### 3.2. PK/PD Analysis

Table 4 shows the achievement rate of the PK/PD targets with linezolid administered as intermittent (AUC_24_/MIC ≥ 80 and 100% T_>MIC_) or continuous (C_ss_ ≥ MIC) infusion. After the standard dose was administered (600 mg/12 h), the PK/PD targets were not reached in many patients, especially for MICs of 2 and 4 mg/L. No differences between patients treated or not with CRRT were detected. When linezolid was administered by continuous infusion, the target (C_ss_ > MIC) was achieved in 100% of the patients for MIC of 1 mg/L, and in more than 85% for the MIC of 2 mg/L.

## 4. Discussion

In this study, we developed a population pharmacokinetic model for linezolid in critically ill patients. Data were best supported by a two-compartment model, in accordance with previously published manuscripts [13,14]. The volume of distribution (45.2 L), which approximated the total body water, was similar to values obtained in other studies in critically ill patients [13,14], and it did not differ from patients with or without CRRT. Moreover, the volume of distribution was similar to that described by Slatter et al. [15] and by MacGowan et al. [16] in healthy volunteers. The moderate lipophilic nature of linezolid may explain the lack of differences in this parameter among critically ill patients and healthy volunteers. Contrary to hydrophilic drugs, whose V is conditioned by the increased extracellular fluid in critically ill patients, the volume of distribution of lipophilic drugs hardly changes in these patients with respect to healthy subjects.

Regarding linezolid elimination, both non-renal and renal clearance were included in the model, the latter being influenced by patients’ Clcr. The inclusion of this covariate in the PK model is controversial. Thus, while some authors found a correlation between Clcr and total clearance [14,17], others concluded that no strong relationship could be demonstrated [13,18]. The poor correlation might be attributable to the use of the Cockcroft–Gault equation to calculate Clcr, which is not accurate enough for critically ill patients [19]. In our study, Clcr was calculated based on creatinine measured in urine, and when it was included as covariate in the model, the IIV of the total clearance decreased from 97.8% to 61.5%. The overall high variability found among patients was consistent with other population PK models performed in critically ill patients, such as that described by Taubert et al. [13] or by Ide et al. [14].

Since linezolid is partially eliminated by CRRT [20], the CL_EC_ was also included in the equation of the total body clearance. The estimation of drug elimination in critically ill patients undergoing CRRT may become complex, since it is influenced by antibiotic-related and CRRT-related characteristics, such as surface area, composition and pore diameter of the membrane, the effluent flow, or the CRRT modality [6]. In this study, the mean Sc value was around 0.8, similar to the unbound protein fraction measured in critically ill patients [17], and did not substantially vary depending on the technique or membrane used. The lack of differences in the Sc depending on the technique has been described previously and might be due to the low molecular weight of the antibiotic [21]. However, the effluent flow justifies differences in CLEC between continuous venovenous hemofiltration (CVVH), CVVHD or CVVHDF. In fact, lower extracorporeal clearance values have been obtained when using CVVH or CVVHD [22,23], as compared to CVVHDF [24], where higher effluent flows are commonly employed. The calculated extracorporeal clearance values in our study (Table 1) were consistent with the aforementioned research works.

It is well-known that, at least in healthy volunteers, linezolid clearance occurs by both renal and hepatic mechanisms (around 30% and 65%, respectively). Liver metabolism occurs by oxidation of the morpholine ring of the drug, without involvement of the cytochrome P450 system [15]. Taking this into consideration, the inclusion in the model of covariates associated with hepatic functionality was analyzed. Despite using the available bilirubin and transaminases data to create three dichotomous covariates, none of them improved the PK model. This outcome might be due to the lack of reliable, economical, and untroublesome parameters of liver function that allow for the prediction of drug metabolic clearance [25,26].

In around one-third of the patients, a peak in the concentration–time curve between 2 and 4 h after dose was detected, which could not be explained with the two-compartment model. As previous studies had demonstrated that linezolid presents biliary excretion [27], we tested a model with enterohepatic circulation; unfortunately, our data were not powerful enough to cope with this model.

In spite of the fact that the IIV estimated with the base model can be explained in part by the Clcr and by the CL_CE_, it is still quite high, as discussed above. Although several demographic and biochemical data were evaluated, unfortunately, we were not able to identify any other variable that could explain the IIV in the PK parameters. The heterogeneity and the limited number of patients may explain that some of the studied covariates resulted in being nonsignificant. However, our study was useful to detect the limitation of the standard dose of linezolid to achieve the PK/PD targets for the clinical breakpoints and to show the advantage of the continuous infusion. In fact, adjustment of linezolid dosing in critically ill patients has been suggested, since recent studies have shown a substantial percentage of subtherapeutic levels [5,6,7,14]. Empirical therapy should cover the clinical breakpoints: in the case of linezolid 2 mg/mL for enterococci (according the Clinical and Laboratory Standard Institute (CLSI) [28]) and 4 mg/L for staphylococci and enterococci (according to the European Committee on Antimicrobial Susceptibility Testing (EUCAST) [29]. In a relevant percentage of patients of our study, for MIC of 2 and 4 mg/L, the PK/PD targets were not reached with the administration of linezolid as intermittent infusion (Table 4), regardless of whether or not they were treated with CRRT.

Different authors have suggested that the administration of a continuous infusion of linezolid could be an alternative option to avoid toxicity problems that could occur when increasing the dose [13,30]. In order to know if the administration of linezolid as a continuous infusion would lead to an improvement of the target attainment, we implemented the study with a new set of patients, who received 50 mg/h of linezolid. Plasma concentrations in the samples from these patients were also used for the external validation of the population model. The selected model was able to predict linezolid plasma concentrations in the patients who received linezolid as continuous infusion. Although statistically significant differences in demographic and biochemical data were observed among patients with short and continuous linezolid infusion, they did not affect the concentration prediction. When comparing the achievement of the PK/PD targets between continuous and intermittent infusion, we observed that it was higher when the patients received linezolid as continuous infusion, regardless of the MIC value, although, only for the MIC of 1 mg/L, the difference was significant. However, it is important to take into account that we have not compared the same information: percent of patients in the case of the intermittent infusion, and percent of samples in the case of continuous infusion (since we had four samples per patient). Therefore, continuous infusion of linezolid seems to be a good option to treat critically ill patients with infections caused by bacteria with MIC values of 2 mg/L. Two of the patients had, at least, one plasma concentration higher than 10 mg/L (concentrations above this value have been associated with side effects), but no sign of toxicity was detected. It should be taken into consideration that this threshold was stated for short infusion, where higher antibiotic concentrations are observed during the dosing interval, and it should not be compared to a constant concentration of 10 mg/L. Thus, further studies are needed to assess the most indicative concentration linked to toxicity for continuous infusion. The major side effect related to linezolid administration is reversible myelosuppression, especially thrombocytopenia. Risk factors associated with this process are chronic liver impairment [31], prolonged duration of linezolid therapy (>14 days), low body weight, or renal failure [32,33].

Therapeutic drug monitoring has been suggested by some authors to optimize linezolid therapy in critically ill patients [18,34]. Our results are in line with this idea. On the one hand, pathophysiological alterations lead to a high inter-individual variability in the pharmacokinetic profile; on the other hand, for the patient undergoing CRRT with or without renal residual function, these factors may have an important impact on the total clearance of the antibiotic and, therefore, on the plasma and tissue concentrations.

## 5. Conclusions

A population pharmacokinetic model for linezolid was developed for critically ill patients. A two-compartment model best described the data. Total body CL was the sum of CL_NR_, CL_R_ (affected by CLcr), and in patients treated with CRRT, the CL_EC_. Our study confirmed that the standard regimen of linezolid may be insufficient to reach the PK/PD target to cover infections caused by pathogens with MICs ≥ 2 mg/L. The administration of linezolid as continuous, instead of intermittent, infusion notably increases the achievement of PK/PD target.

## Figures and Tables

**Table 1:** Hospital, demographic and biochemical data, and APACHE II health score (Acute Physiology and Chronic Health Evaluation II) of the 40 patients. AUH: Araba University Hospital; DOUH: Doce de Octubre University Hospital; JUH: Joan XXIII University Hospital; CRRT: continuous renal replacement therapies; BMI: body mass index; Clcr: creatinine clearance; GOT: glutamate oxaloacetate transaminase; GPT: glutamate pyruvate transaminase; CL_EC_: extracorporeal clearance; CVVHDF: continuous venovenous hemodiafiltration; CVVHD: continuous venovenous hemodialysis.

	No CRRT		CRRT	
Patient Characteristic	n	Median (Range)	n	Median (Range)
**Hospital**				
AUH	17		9	
DOUH	0		13	
JUH	0		1	
**Demographic Data**				
Age (years)		72 (22–85)		68 (37–79)
Gender (M/F)	13/4		16/7	
Body weight (kg)		71 (60–95)		74 (55–110)
Height (m)		1.70 (1.60–1.85)		1.69 (1.53–1.85)
BMI (kg/m^2^)		24.5 (20.8–31.3)		25.9 (21.2–33.1)
**Biochemical Data**				
Clcr (mL/min)		71.2 (11.0–179.5)		6.0 (0.0–45.6)
Creatinine (mg/dL)		0.80 (0.40–2.10)		1.15 (0.56–2.60)
Glucose (mg/dL)		144 (73–187)		140 (69–210)
Hemoglobin (g/dL)		9.85 (7.00–15.50)		8.50 (6.70–11.40)
Hematocrit (%)		30.5 (20.0–46.0)		26.4 (18.7–34.7)
Albumin (g/dL)		2.8 (1.9–4.0)		2.2 (1.7–3.6)
Total proteins (g/dL)		5.8 (4.2–7.4)		5.2 (2.7–7.3)
Bilirubin (mg/dL)		0.65 (0.20–1.30)		0.80 (0.12–2.60)
GPT (U/L)		25 (6–340)		50 (5–570)
GOT (U/L)		33 (16–330)		43 (8–675)
**APACHE II**		16 (11–36)		22 (16–34)
CL_EC_ (L/h)			23	2.51 (0.79–3.09)
CVVHDF			18	2.61 (0.79–3.09)
CVVHD			5	1.06 (1.00–2.73)

**Table 2:** Demographic and biochemical data and APACHE II health score of the patients used for the validation of the model. BMI: body mass index; Clcr: creatinine clearance; GOT: glutamate oxaloacetate transaminase; GPT: glutamate pyruvate transaminase.

Patient Characteristic	n	Median (Range)
**Demographic Data**		
Age (years)		60 (24–84)
Gender (M/F)	4/7 *	
Body weight (kg)		80 (70–115)
Height (m)		1.67 (1.65–1.80)
BMI (kg/m^2^)		28.5 (24.2–35.5)
**Biochemical Data**		
Clcr (mL/min)		111 (45–240) *
Creatinine (mg/dL)		0.70 (0.50–1.10) *
Glucose (mg/dL)		136 (114–217)
Hemoglobin (g/dL)		10.3 (8.2–11.7)
Albumin (g/dL)		3.0 (2.5–3.6) *
Total proteins (g/dL)		5.4 (5.3–7.4) *
Bilirubin (mg/dL)		0.70 (0.50–2.10)
GPT (U/L)		37 (18–220)
GOT (U/L)		25 (16–74)
**APACHE II**		16 (6–25) *

* Significant differences (*p < 0.05*) with respect to the patients used for the development of the model (Table 1).

**Table 3:** Base and final population pharmacokinetic model estimates, shrinkage ^a^ values, and bootstrap results after short-term intravenous infusion.

Parameter	Base Model	Final Model	Bootstrap Median (5th–95th Percentile)
	Estimate, RSE (%)	Estimate, RSE (%)	
CL (L/h) ^b^ = (CL_NR_ + CL_R_)+ CL_EC_	5.59 (13) + CL_EC_		
CL_NR_		2.62 (18)	2.65 (2.02–3.65)
CL_R_ = θ × (Clcr/44)		4.35 (19)	4.33 (2.99–5.84)
CL_EC_^c^ = Sc × Qef			
V_1_ (L) ^d^	16.1 (20)	16.2 (14)	16.6 (11.7–24.4)
Q (L/h)	72.3 (18)	71.7 (14)	69.5 (40.4–92.0)
V_2_ (L)	29.1 (8)	29.0 (7)	28.6 (23.0–32.6)
IIV__CL_ (%)	98.7 (10)	61.5 (9)	59.3 (48.9–69.4)
IIV__V1_ (%)	66.6 (20)	65.9 (17)	62.4 (37.7–91.6)
Residual error_additive (mg/L)	0.260 (24)	0.266 (24)	0.267 (0.156–0.464)
Residual error_proportional (%)	0.160 (9)	0.159 (19)	0.157 (0.122–0.183)

^a^ CLηsh = 1%; V_1_ηsh = 18%; εsh = 11%; ^b^ base model: CL = (5.59 + (Sc × Qef)) × exp(η1); final model: CL = (2.62 + (4.35 × (Clcr/44) + Sc × Qef)) × exp(η1); ^c^ CL_EC_ was included as the value calculated for each patient (median 2.51, range: 0.79–3.09), only for those undergoing CRRT; ^d^ base model: V_1_ = 16.1 × exp(η2); final model: V_1_ = 16.2 × exp(η2). CL: total body clearance; CL_NR_: non-renal clearance; CL_R_: renal clearance; CL_EC_: extracorporeal clearance; Clcr: creatinine clearance; Sc: Sieving coefficient; Qef: effluent flow; V_1_: apparent volume of distribution of the central compartment; V_2_: apparent volume of distribution of the peripheral compartment; IIV: inter-individual variability; RSE: relative standard error; ηsh: shrinkage value for a parameter; εsh: shrinkage value for the residual error.

**Table 4:** Achievement rate of the PK/PD targets with linezolid administered as intermittent or as continuous infusion to critically ill patients.

	600 mg/12 h 30 min Infusion						50 mg/h Continuous Infusion	
	AUC_24_/MIC ≥ 80			100% T > _MIC_			C_ss_ ≥ MIC	
MIC (mg/L)	CRRT (*n* = 23)	No CRRT (*n* = 17)	*p*-value ^a^	CRRT (*n* = 23)	No CRRT (*n* = 17)	*p*-value ^a^	*n* = 11 44 plasma samples	*p*-value ^b^
1	22 (96%)	13 (76%)	0.07	19 (83%)	13 (76%)	0.63	44 (100%)	0.009
2	12 (52%)	11 (65%)	0.43	15 (65%)	12 (71%)	0.72	38 (86%)	0.15
4	0	1 (6%)	0.24	7 (30%)	5 (29%)	0.94	22 (50%)	0.15

^a^ CRRT vs. no CRRT; ^b^ respect to 100% T > MIC and no CRRT. Significant differences if *p* < 0.05.
